# *Leonotis nepetifolia* Transformed Root Extract Reduces Pro-Inflammatory Cytokines and Promotes Tissue Repair In Vitro

**DOI:** 10.3390/ijerph20064706

**Published:** 2023-03-07

**Authors:** Przemysław Sitarek, Tomasz Kowalczyk, Tomasz Śliwiński, Sophia Hatziantoniou, Nikolitsa Soulintzi, Rafal Pawliczak, Joanna Wieczfinska

**Affiliations:** 1 Department of Biology and Pharmaceutical Botany, Medical University of Lodz, Muszynskiego 1, 90-151 Lodz, Poland; 2 Department of Molecular Biotechnology and Genetics, University of Lodz, Banacha 12/16, 90-237 Lodz, Poland; 3 Laboratory of Medical Genetics, Faculty of Biology and Environmental Protection, University of Lodz, Pomorska 141/143, 90-236 Lodz, Poland; 4 Laboratory of Pharmaceutical Technology, Department of Pharmacy, School of Health Sciences, University of Patras, 26504 Patras, Greece; 5 Department of Immunopathology, Medical University of Lodz, Zeligowskiego 7/9, Bldg 2, Rm 177, 90-752 Lodz, Poland

**Keywords:** *Leonotis nepetifolia* root extract, transformed root extract, inflammation, cytokines, airway cells

## Abstract

Inflammation is closely related to asthma and its defining feature: airway remodeling. The aim of this study was to determine the effects of extracts of normal (NR) and transformed (TR) *Leonotis nepetifolia* roots on respiratory cells and against the gingival epithelium. Extracts from NR and TR roots were added to lung fibroblast, bronchial epithelial and gingival fibroblast cell lines, in the presence of HRV-16 infection, to determine their impact on inflammation. The expression of inflammatory cytokines (IL-6, IL-1β, GM-CSF and MCAF) as well as total thiol contents were assessed. The TR extract inhibited rhinovirus-induced IL-6 and IL-1β expression in all tested airway cells (*p* < 0.05). Additionally, the extract decreased GM-CSF expression in bronchial epithelial cells. The tested extracts had positive effects on total thiol content in all tested cell lines. The TR root extract demonstrated wound healing potential. While both tested extracts exhibited anti-inflammatory and antioxidative effects, they were stronger for the TR extract, possibly due to higher concentrations of beneficial metabolites such as phenols and flavonoids. Additionally, wound healing activity was demonstrated for the TR root extract. These results suggest that TR root extract may become a promising therapeutic agent in the future.

## 1. Introduction

Among respiratory disorders, such as COPD, asthma, lung cancer and sleep apnea, asthma is growing in importance as a global public health problem with increasing mortality rates [1]. The condition involves airway remodeling characterized by subepithelial fibrosis, thickening of the airway walls, angiogenesis, an increase in the mass of smooth muscles as well as enlargement of the mucous glands. The immediate consequence of airway remodeling is persistent reactivity and irreversible airway obstruction, leading to chronic and persistent asthma [2]. During asthma exacerbations, inflammatory cascades stimulate the immune system, with increased production of T helper (Th) 2 lymphocytes; these are characterized by the release and subsequent imbalance of Th1 and Th2 cytokines. It also results in increased secretion of Th2 cytokines such as interleukin (IL)-4, IL-5 and IL-13, which cause airway inflammation and consequently airway infiltration, eosinophil activation and differentiation, immunoglobulin E (IgE) production and mucus secretion [3].

Furthermore, many inflammatory cytokines are involved in asthma-connected airway inflammation. It has been suggested that IL-1β, which is a potent proinflammatory cytokine, may induce fibrogenic markers in airway fibroblasts, resulting in transition of the fibroblasts to myofibroblasts [4]. Additionally, IL-6 is an important, asthma-involved cytokine. Patients with asthma have been reported to have elevated blood levels of IL-6. More significantly, IL-6 levels in bronchoalveolar lavage fluid (BALF) have been found to be higher in active asthmatics as compared to stable asthmatics, non-asthmatic patients undergoing mechanical ventilation and healthy non-smokers [5].

Pharmacological treatment of asthma is usually based around corticosteroids, β-agonists and leukotriene receptor antagonists [6]. Many of these drugs have a variety of side effects such as oral candidiasis, cataracts, hoarseness, reduced bone density in adults, glaucoma, increased heart rate, anxiety, rashes or dizziness. An interesting alternative is presented by plant-derived extracts and pure compounds, mainly due to their availability for low-income populations and also relatively low toxicity. Numerous studies show that both extracts and their purified constituent compounds can modulate interleukin levels to control and reverse asthma exacerbations in vivo and in vitro [7,8,9]. Qu et al. showed that *Astragalus* extract improved asthma airway remodeling by inhibiting the expression of the TGF-β1/Smad signaling pathway in mice [10]. In turn, Peng et al. demonstrated that Platycodin D, a triterpenoid saponin extracted from *Platycodon grandiflorus* root, alleviated airway remodeling and inflammation in asthmatic mice, which might be related to downregulating the phosphorylated proteins in the MAPK/NF-κB signaling pathway [11]. Our previous studies revealed that AtPAP1 TR root extract from *Leonurus sibiricus* may affect the remodeling process by inhibiting the HRV16-induced expression of arginase I, MMP-9 and TGF-β in two fibroblast, WI-38 and HFL1, lines [12].

An interesting plant with multidirectional antimicrobial, anti-inflammatory, antioxidant, wound healing, analgesic and anticancer activities is *Leonotis nepetofolia* (L.) R. Br, a member of the *Lamiaceae* family found in tropical regions of Africa and southern India. Numerous studies show that a range of alkaloids, terpenoids, phenolic acids or flavonoids may be responsible for its biological properties [11,12,13,14,15]. *Leonotis nepetifolia* extract may act by increasing intracellular glutathione (GSH), reducing reactive oxygen species (ROS) levels and preventing Ca^2+^ influx when ROS levels are high, thus providing protection against oxidative damage [13,14,15]. The biological activity of the extracts can be further enhanced through green biotechnology strategies such as induction of hairy roots, formed by infection with the bacterium *Rhizobium rhizogenes*. These can be an excellent alternative for obtaining secondary metabolites in large quantities due to the high growth rate and the possibility of obtaining large quantities of biomass in a shorter period of time; in addition, this approach also negates the adverse environmental impact of harvesting conventional plant material. Various biological studies demonstrate that the extracts obtained from transformed roots are stronger than those from normal roots, which could be due to higher accumulation of selected bioactive metabolites [16,17].

This study demonstrates the effect of unique extracts obtained from normal and transformed *Leonotis nepetifolia* roots, derived in vitro, on inflammation and airway remodeling in different cell lines; these effects appear to act by altering the expression of virus-induced inflammatory cytokines at the gene and protein level. The work also analyzes the influence of the extracts on gingival cells and provides an insight into their anti-inflammatory or wound healing properties.

## 2. Material and Methods

### 2.1. Plant Material and Extract Preparation from Leonotis nepetifolia Roots

Two different extracts from normal (NR) and transformed (TR) roots of *Leonotis nepetifolia* were used in the study. Briefly, lysophilized and powdered plant materials were extracted with aqueous methanol using an ultrasonic bath. The two extracts were then filtered, combined and evaporated under reduced pressure, followed by lyophilization. The exact procedure is described elsewhere [17].

### 2.2. Cell Cultures

WI-38 was acquired from (Sigma-Aldrich, St. Louis, MO, USA). The cells were cultured in EMEM medium with 10% fetal bovine serum, 2 mM L-glutamine, 1% non-essential amino acids and standard Penicillin-Streptomycin solution. The NHBE epithelial cell line was obtained from Lonza (Lonza Inc., Walkersville, MD, USA). It was cultivated in BEGM (BulletKit for Bronchial Epithelial Cell Growth Medium) (Lonza Walkersville Inc., Walkersville, MD, USA). Human gingival fibroblasts were purchased from the American Type Culture Collection (ATCC), and cultured in Dulbecco’s Modified Eagle Medium (DMEM), with 10% fetal bovine serum, 100 U/mL penicillin and 0.1 mg/mL streptomycin. 

While the use of respiratory cells seems obvious in the context of asthma and airway remodeling, gingival cells were also included to investigate the anti-inflammatory effects of the extracts and to highlight differences between bronchial and gingival epithelia during treatment. All of the experiments (n = 6) were performed after reaching 80–90% confluence (passage three to ten) by the cells. The viability of the cells was evaluated by Presto Blue (BD Pharmingen, Franklin Lakes, NJ, USA), the absorbance was measured at 570 nm.

### 2.3. Virus Preparation and Cell Infection

Human Rhinovirus (HRV) 16 was made available by the European Collection of Authenticated Cell Cultures (ECACC, Salisbury, UK). A cytopathic impact was observed after infecting HeLa cells with an MOI of 1, as indicated by the literature [18,19]. The target cells were infected by 50 L of HRV16 or vehicle (medium) and incubated for 24 h (33 °C, 5% CO_2_).

### 2.4. Experimental Procedure 

The cultures were treated with the HRV-16 virus for 24 h (33 °C, 5% CO_2_). They were then treated with 400 µg/mL of normal or modified root extract from *Leonotis nepetifolia* for 24 h at 37 °C with 5% CO_2_. The control samples were treated with medium only. 

### 2.5. RNA Isolation and cDNA Synthesis

Total RNA was isolated utilizing a Total RNA mini kit (A&A Biotechnology, Gdynia, Poland), then purified and stored at −80 °C. Reverse transcription using 1 μg of RNA was performed using a High-Capacity cDNA kit (Applied Biosystems, Foster City, CA, USA) according to the manufacturer’s instructions. 

### 2.6. Gene Expression Analysis

The changes in the expression of *IL-6*, *IL-1β*, *GM-CSF* and *MCAF* were determined by qPCR. TaqMan gene expression assays were used for the selected genes: IL-6—Hs00174131_m1, IL-1β—Hs01555410_m1, GM-CSF—Hs99999044_m1, MCAF—Hs00234140_m1 (Life Technologies, Carlsbad, CA, USA). Each sample was measured in triplicate using the TaqMan analyzer and gene expression calculated by the 2^−ΔΔCt^ method. The results were normalized to an endogenous reference gene (β-actin—Hs99999903_m1), and the fold change in mRNA expression was calculated.

### 2.7. ELISA Test

The concentrations of IL-6, IL-1β, GM-CSF and MCAF were determined by ELISA using a commercial MyBiosource kit (San Diego, CA, USA), according to the manufacturer’s protocol.

### 2.8. Colorimetric Thiol Detection (R-SH)

Total thiol content was measured using 5,5′-dithiobis-(2-nitrobenzoic) acid (DTNB), known as Ellman’s reagent (Sigma, St. Louis, MO, USA) [20]. Briefly, 1 mL of PBS and 1 mM of EDTA (pH 7.5) was added to 30 µL of a sample and incubated at room temperature for 30 min. Subsequently, the absorbance was read at 412 nm, indicating the amounts of 5-thio-2-nitrobenzoic acid (TNB) formed. Control samples were prepared without DTNB or protein and these were run simultaneously.

### 2.9. Cell Migration (Scratch Test, Wound Healing) Assay

The influence of the extracts on wound healing was assessed based on NIH3T3 murine fibroblast migration. NIH3T3 fibroblasts from the second to sixth passage were defrosted and cultured in T75 flasks in Dulbecco’s Modified Eagle’s Medium (DMEM) with 10% fetal bovine serum (FBS) and 1% antibiotics Penicillin and Streptomycin (P/S) until confluence. Following this, 5 × 10^4^ NIH3T3 cells were seeded in 24-well culture plates, where they were grown to a confluent (80%) cell monolayer in 1 mL of DMEM media with 10% FBS and 1% antibiotics P/S. They were then cultivated for three hours in starvation medium (DMEM, 1% antibiotics P/S).

A 200 L pipette tip was used to scratch the confluent cell culture in the midline to simulate a wound. After being washed with PBS, different concentrations of TR root extract were filtered through a sterile syringe filter (0.22 m pore size, PES membrane, non-pyrogenic Thermo scientific), aliquoted in DMEM media (DMEM, 0.1% BSA, 1% P/S), and added into the wells. The width of the wound was measured using a micro-camera (Soft plus skin analyzer, Callegari, Italy) at three time points: immediately after scratching (time 0), then at 24 and 48 h. The same frame was measured each time.

The percentage of wound recovery (*WR*%) at any time point (*t*) was calculated using Equation (1), where *SW*_0_ and *SW_t_* are the widths of the scratch at time 0 and t, respectively.

(1) $$WR\%=\frac{SW_{0}-SW_{t}}{SW_{0}}\times 100$$

A positive control was created with 2% FBS and a negative control was created with sample vehicle (DMEM, 0.1% BSA, 1% P/S). The positive controls displayed a small positive proliferating/migrating capacity, but the negative controls did not show any changes. Both controls and samples were tested twice.

### 2.10. Statistical Analyses

The results were analyzed using Statistica (StatSoft, Tulsa, OK, USA). Homogeneity of variance was confirmed using Levene’s test and data distribution using the Shapiro–Wilk test. Significant differences were identified between groups using ANOVA and *p* values of 0.05 or below were considered significant.

## 3. Results

### 3.1. Phytochemical Analysis of NR and TR Root Extract from Leonotis nepetifolia

Different phenolic and flavonoid contents were found in the two tested extracts, with higher amounts of phenols and flavonoids observed in the TR extract. The dominant compounds in both tested extracts were (+)-catechin, p-coumaric acid, m-coumaric acid and rosmarinic acid [17].

### 3.2. Cell Viability

**Figure 1:**
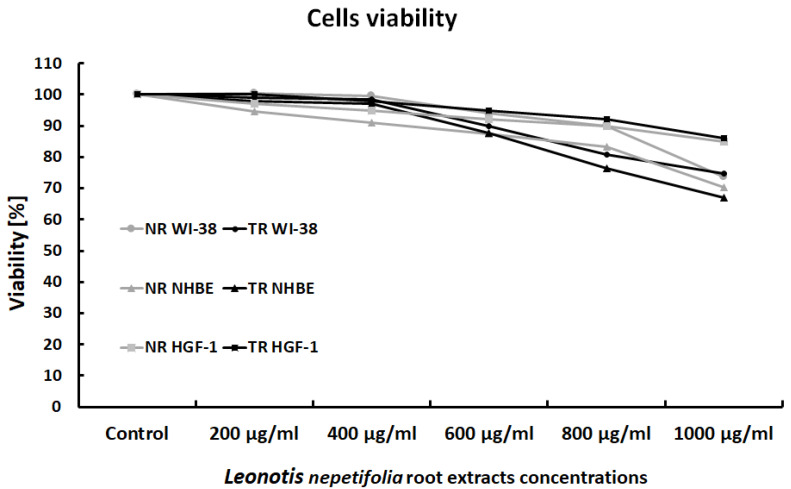
Viability of WI-38, NHBE and HGF-1 cells after exposure to normal (NR) and transformed (TR) *Leonotis nepetifolia* root extracts. Cell viability was assessed after 24 h. The values represent mean viability.

*Leonotis nepetifolia* root extracts showed no cytotoxicity towards any of the cell lines used. Gingival fibroblasts (HGF-1) appeared to be the least sensitive to the effects of the extracts. No statistically significant differences were observed between cell viability after incubation with normal (NR) or transformed (TR) extracts of *Leonotis nepetifolia* (Figure 1).

### 3.3. The Effects of NR and TR on Gene Expression

**Figure 2:**
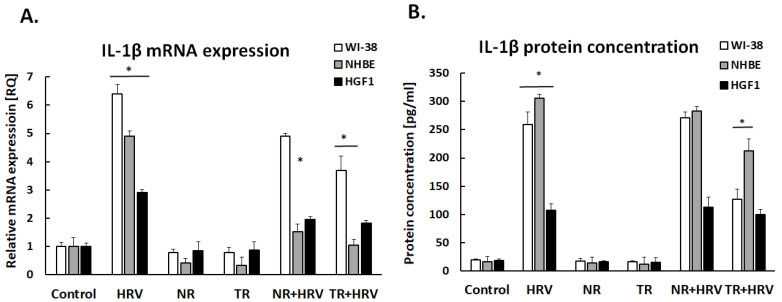
IL-1β mRNA (**A**) and protein (**B**) expression after treatment with normal (NR) and transformed (TR) *Leonotis nepetifolia* root extracts following HRV infection. Data presented as mean ± SEM, * *p* < 0.05.

Rhinovirus induced interleukin-1β expression in both epithelial airway cells and gingival fibroblasts (Figure 2). Both the NR and TR extracts decreased HRV-induced interleukin-1β expression in both fibroblasts and respiratory epithelial cells.

**Figure 3:**
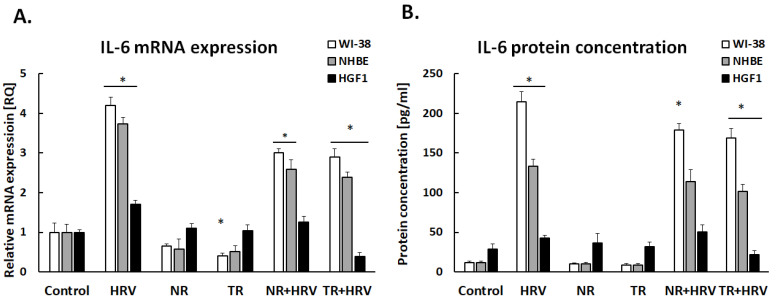
*Leonotis nepetifolia* extracts from normal (NR) and transformed (TR) roots, decreased virus-induced IL-6 expression on mRNA (**A**) and protein (**B**) levels. Data presented as mean ± SEM, * *p* < 0.05.

IL-6 expression was significantly increased after HRV stimulation (*p* < 0.05). Both the NR and TR extracts decreased virus-induced IL-6 expression, but only in airway cells (NHBE and WI-38, *p* < 0.05, Figure 3). 

**Figure 4:**
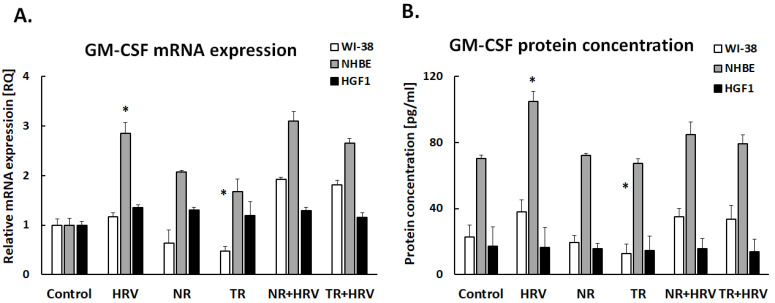
The effect of the *Leonotis nepetifolia* NR and TR root extracts and viral infection (HRV-16) on the expression of GM-CSF on mRNA (**A**) and protein (**B**) levels in human cells. Data presented as mean ± SEM, * *p* < 0.05.

**Figure 5:**
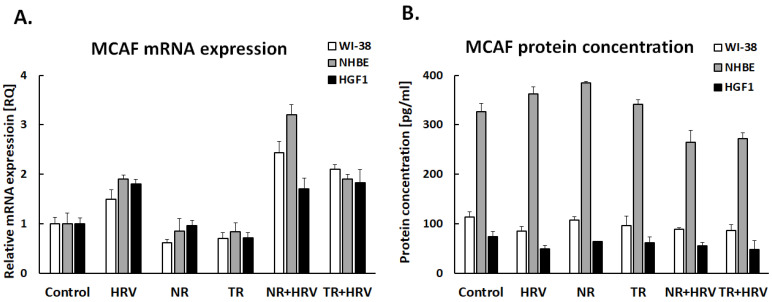
Expression of MCAF in mRNA (**A**) and protein (**B**) levels after treatment with (NR) and (TR) *Leonotis nepetifolia* root extracts. Data presented as mean ± SEM, * *p* < 0.05.

Interestingly, none of the tested extracts had any significant effect on GM-CSF expression in lung fibroblasts or gingival cells (Figure 4). The TR extract reduced GM-CSF expression in bronchial epithelial cells, but not HRV-induced expression. The tested extracts had no effect on MCAF expression (*p* > 0.05, Figure 5). 

### 3.4. The Effects of NR and TR Root Extracts on Total Thiol Content

**Figure 6:**
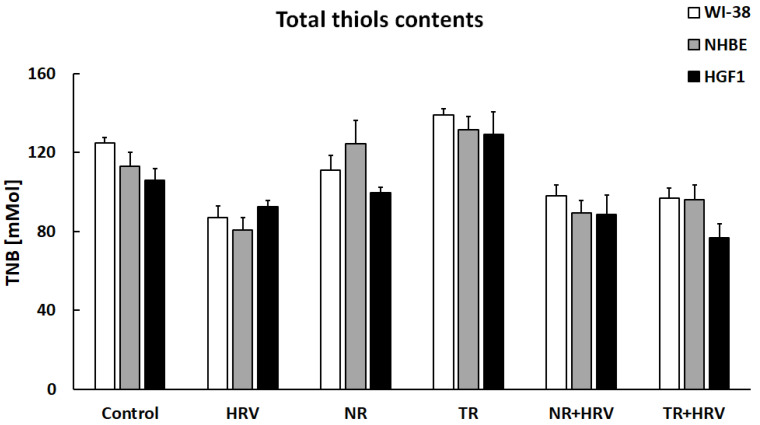
The lack of effect of NR or TR extracts on total thiol groups in WI-38, NHBE and HGF-1 cells. Data presented as mean ± SEM.

Total thiol content (R-SH) was significantly reduced by HRV infection in both airway cell lines and in the gingival cells. TR root extract significantly increased thiol concentration in all cell types (*p* < 0.05) and NR extract in bronchial epithelial cells. No significant changes in total thiol content were seen in any treated cell type in the presence of HRV (Figure 6).

### 3.5. Scratch Test 

**Figure 7:**
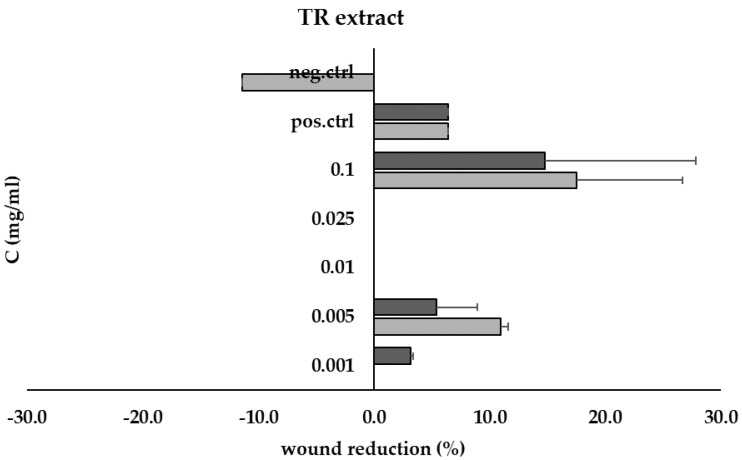
Wound recovery (% scratch reduction) at 24 h (■) and 48 h (■) for TR root extract at concentrations ranging from 0.001 mg/mL to 0.1 mg/mL, compared to positive (pos.ctrl) and negative (neg.ctrl) controls.

The TR root extract used for the scratch test had demonstrated stronger biological properties in previous studies. The TR root extract was tested at a range of concentrations from 0.001 mg/mL to 0.1 mg/mL. The results are depicted in Figure 7. The TR extract demonstrated migratory/proliferative properties from a minimum concentration of 0.001 mg/mL. This effect was noted after 24 h of incubation and was similar to the effect of the positive control (*p* > 0.05).

## 4. Discussion

Although the ability of rhinovirus to trigger airway remodeling remains unclear, it has been linked to allergen-induced asthma exacerbations in people as well as transitory lung inflammation [21,22]. IL-1β is a powerful pro-inflammatory cytokine that plays important roles in systemic disorders including inflammatory bowel disease, type 2 diabetes and rheumatoid arthritis, as well as the early response to infection [23]. IL-1β acts via the IL-1 receptor, activating AP-1 and NF-κB [23]. 

Our findings indicate the HRV induced IL-1β expression in all cell types, as noted previously [24,25]; however, it is unclear whether different cell types use identical mechanisms to increase IL-1β expression. It has been demonstrated that IL-1, IL-6 and IL-8 production is increased in respiratory progenitor cells after HRV-1β infection, similar to endogenous airway epithelial cells [26]. More importantly, the TR *Leonotis nepetifolia* roots extract reduces HRV-induced IL-1β expression, but not the NR extract; this indicates that the TR extracts are more effective in modulating the expression of IL-1β, and that transformation is a valuable tool for increasing the concentrations of beneficial ingredients. The IL-1β receptor antagonist anakinra has been found to limit the growth of Th2/Th17 cells in vitro [27]. Anakinra was also found to decrease airway neutrophilia in a mouse model of asthma. However, no clinical trials have tested the compound as a medication in asthmatic patients.

It is possible that our positive findings may be caused by the phenols and flavonoids found in the tested extracts; *L. nepetifolia* TR root extract is known to have greater antioxidant potential since it includes higher amounts of phenols and flavonoids, which possess antioxidative and ROS scavenging properties [17,28]. Moreover, polyphenols also have anti-inflammatory properties [29,30]. Therefore, the phenols and flavonoids found in the examined extracts may be responsible for modulating the expression of the pro-inflammatory agents in this study. 

Th literature reports that *Leonotis nepetifolia* extracts contain the anti-inflammatory flavonoid cirsiliol (3′,4′,5-trihydroxy-6,7-dimethoxyflavone) [31,32,33], in turn, our earlier study reported high content of (+)-catechin, p-coumaric acid, m-coumaric acid and rosmarinic acid in the transformed root extract of *Leonotis nepetifolia*, which also may have an anti-inflammatory effect [17]. This may explain our results, since the TR extracts more effectively inhibited both IL-1β and IL-6 expression—probably due to higher concentrations of anti-inflammatory factors. In addition, IL-6 expression was also reduced by the normal (NR) extract, confirming that the plant naturally has anti-inflammatory effects; however, this effect was only observed in respiratory cells. In gingival cells, TR treatment significantly reduced IL-6 level, but no effect was observed for NR, confirming that transformation enriches the flavonoid composition of the extract.

The flavonoids are a large family of natural phenols that contain thousands of different chemicals, including flavonols, flavones, flavan-3-ol (catechins), flavanones, anthocyanidins, and isoflavonoids. It is crucial to remember that phytochemical combinations might affect the biological impact that is anticipated to be induced by the individual chemicals [34,35]. 

Flavonoids are known to have good anti-inflammatory properties. The structure of flavonoids plays an important role in anti-inflammatory activity. For example *Betulaceae*, *Fabaceae*, *Asteraceae* and *Apiaceae* families are rich in quercetin, which extincts free radicals formed during cellular metabolism [36]; *Asphodelaceae* and *Scrophulariaceae* families contain luteolin, which presents anticancer properties and is associated with the induction of apoptosis and inhibition of cell proliferation, metastasis and angiogenesis [37], while the *Fabaceae* family is abundant in kaempferol, isoflavones and flavanols, which are known to act as antioxidants and anticancer and anti-inflammatory agents in various contexts [38,39]. 

The catechins are a class of polyphenols that also act as free radical scavengers; indeed, catechin is a highly effective radical scavenger [40,41]. Treatment with cirsiliol has no effect on interaction between IL-6 and its receptor; however, it inhibits IL-6-induced inflammatory marker gene expression levels, including CRP, IL-1β via Jak2 phosphorylation. It is thus possible that cirsiliol can inhibit IL-6-induced cellular signaling by modulating Jak2 phosphorylation, which may be useful as a treatment for inflammatory illnesses linked to IL-6 [31].

The multifunctional cytokine granulocyte-macrophage colony-stimulating factor (GM-CSF) controls inflammatory reactions in a variety of autoimmune and inflammatory diseases [42]. GM-CSF is produced by epithelial cells and fibroblasts under the influence of IL-17. Kim et al. showed that reduced production of IL-17 and GM-CSF may be one of the mechanisms contributing to reducing inflammatory cell infiltration and preventing airway remodeling [43].

GM-CSF levels may be important in gingival cells to distinguish between the inflammatory state (periodontitis) in its early and later phases [44]. Furthermore, GM-CSF can alter the in vitro expression of a few membrane molecules of gingival fibroblasts, suggesting that it may control the distinctive biological processes of gingival tissue in vivo [45]. In our study, we showed that GM-CSF expression was significantly reduced in lung fibroblasts, while remaining unaffected in the other cells studied. This effect was achieved by the action of *Leonotis nepetifolia* transformed root extract. Interestingly, GM-CSF expression was not increased by HRV, suggesting that this factor is not important in the pathway of infection with this virus. However, given that GM-CSF is a factor involved in inflammation, its downregulation by the tested extract seems to be a positive effect [46].

Thiols (R-SH), which are substances with sulfhydryl groups (-SH), are non-enzymatic antioxidants that are particularly significant since their measurement presents an indirect picture of the antioxidant defense.

The TR root extract produced a stronger effect than NR. *L. nepetifolia* transformed root extract, increasing the thiol content in each of the cell lines tested, including in the gingival epithelium. Interestingly, the changes in gene expression occurring in these cells were less significant than those in airway cells. In line with our hypothesis, the transformed root extracts contain higher levels of phenols and flavonoids, which have antioxidant properties [47,48]. Therefore, this plant appears to be a valuable producer of beneficial substances that may find a place in future therapies.

In addition, the TR extract was found to have a positive effect on wound healing in the scratch test. The wound healing mechanism is essential to recover lost tissue and maintain tissue homeostasis, however, it is a complex mechanism and various plants traditionally used in medicine have been validated for their wound healing properties [49]. Our studies are consistent with Nithya et al. who showed that a wound treated with *Leonotis nepetifolia* leaf extract healed significantly faster, as indicated by an improvement in contraction rate and shortening of the epithelization period in rats [50]. However, Freiesleben et al. did not identify any wound healing potential after treatment with *Leonotis nepetifolia* plant extract [51]. We suppose that these differences may result from the specificity of the extract used, as well as the active compounds contained in it. Our extract was characterized by a high content of phenolic acids and flavonoids.

## 5. Conclusions

The obvious limitations of our study are that it is performed on cell lines without confirmation on an animal model; however, our findings not only compare two different extracts, but also demonstrate their effects on different cell types. The compounds contained in the transformed *Leonotis nepetifolia* root extract appear to have anti-inflammatory potential that can be further enhanced by biotechnology techniques. In addition, TR treatment ameliorated the action of HRV, which contributes to the development of airway remodeling known to play a significant role in asthma. As the tested extract downregulates the expression of highly pro-inflammatory IL-6 and IL-1β, it may merit potential consideration in therapy for inflammation-related diseases and hence further study. Additionally, TR root extract may play an important role as a therapeutic agent in wound therapy.

## Data Availability

The data presented in this study are available on request from the corresponding author. The data are not publicly available due to restrictions eg privacy or ethical.
